# Cost-Effectiveness Analysis of Biomarker-Guided Treatment for Metastatic Gastric Cancer in the Second-Line Setting

**DOI:** 10.1155/2020/2198960

**Published:** 2020-02-17

**Authors:** Brianna Lauren, Sassan Ostvar, Elisabeth Silver, Myles Ingram, Aaron Oh, Lindsay Kumble, Monika Laszkowska, Jacqueline N. Chu, Dawn L. Hershman, Gulam Manji, Alfred I. Neugut, Chin Hur

**Affiliations:** ^1^Columbia University Medical Center, New York, NY, USA; ^2^Massachusetts General Hospital, Boston, MA, USA; ^3^Columbia University Irving Cancer Research Center, New York, NY, USA

## Abstract

**Background:**

The 5-year survival rate of patients with metastatic gastric cancer (GC) is only 5%. However, trials have demonstrated promising antitumor activity for targeted therapies/immunotherapies among chemorefractory metastatic GC patients. Pembrolizumab has shown particular efficacy among patients with programmed death ligand-1 (PD-L1) expression and high microsatellite instability (MSI-H). The aim of this study was to assess the effectiveness and cost-effectiveness of biomarker-guided second-line GC treatment.

**Methods:**

We constructed a Markov decision-analytic model using clinical trial data. Our model compared pembrolizumab monotherapy and ramucirumab/paclitaxel combination therapy for all patients and pembrolizumab for patients based on MSI status or PD-L1 expression. Paclitaxel monotherapy and best supportive care for all patients were additional comparators. Costs of drugs, treatment administration, follow-up, and management of adverse events were estimated from a US payer perspective. The primary outcomes were quality-adjusted life years (QALYs) and incremental cost-effectiveness ratios (ICERs) with a willingness-to-pay threshold of $100,000/QALY over 60 months. Secondary outcomes were unadjusted life years (survival) and costs. Deterministic and probabilistic sensitivity analyses were performed to evaluate model uncertainty.

**Results:**

The most effective strategy was pembrolizumab for MSI-H patients and ramucirumab/paclitaxel for all other patients, adding 3.8 months or 2.0 quality-adjusted months compared to paclitaxel. However, this strategy resulted in a prohibitively high ICER of $1,074,620/QALY. The only cost-effective strategy was paclitaxel monotherapy for all patients, with an ICER of $53,705/QALY.

**Conclusion:**

Biomarker-based treatments with targeted therapies/immunotherapies for second-line metastatic GC patients substantially improve unadjusted and quality-adjusted survival but are not cost-effective at current drug prices.

## 1. Introduction

Gastric cancer (GC) is the fifth most common cancer worldwide and the third leading cause of cancer-related mortality, with a 30% five-year survival rate for all stages [1, 2]. In addition, 25% of GC patients present with advanced disease, and another 25%–50% progress to metastatic disease [3]. The prognosis is especially poor for nonresponders to first-line (1L) chemotherapy, who face a challenging decision between supportive/palliative care and more aggressive intervention with taxanes, antiangiogenic therapy, or PD-1 blockade. Insurance claims data suggest that, as of 2012, a majority (54.5%) of GC patients who progress on 1L treatment go on to receive a second line of chemotherapy [4]. As novel therapies are incorporated into the second- and third-line (2L, 3L) treatment landscapes, further analysis is warranted to optimize quality-adjusted life years (QALYs) and determine the impact of personalized, biomarker-based treatment on the effectiveness and cost-effectiveness of treatment.

The 2018 National Comprehensive Cancer Network (NCCN) guidelines for treatment of chemorefractory, metastatic, or recurrent gastric cancer include the anti-PD-1 agent pembrolizumab (PEM), the VEGFR-2 antagonist ramucirumab (RAM), and a variety of taxanes, such as paclitaxel (PAC) monotherapy and its combination with RAM (RAM/PAC). Recommendations of RAM and PEM were primarily based on the outcomes of the RAINBOW and KEYNOTE-059 trials, respectively, and studies by Le et al. on deficient mismatch repair (dMMR) tumors (2015, 2017) [5–9]. The guidelines recommend PEM as 2L treatment for GC patients with high microsatellite instability (MSI-H) or deficient mismatch repair (dMMR) status and as 3L treatment for GC patients with positive PD-L1 expression using a 1% combined positive score (CPS) threshold [5].

The KEYNOTE-059 and KEYNOTE-061 trials showed promising results of durable response to PEM. Among PD-L1 + patients on PEM, median overall survival (OS) was 9.1 months and median duration of response was 15.7 months, with a range exceeding 23 months [10]. PAC monotherapy and RAM/PAC combination therapy showed similar OS rates, but much lower median durations of response (2.8 months and 4.4 months, respectively) [7]. This warrants further analysis to determine if durability of response makes PEM a cost-effective therapy.

Personalized or biomarker-based treatment may be crucial to the effectiveness and cost-effectiveness of the treatment landscape for advanced GC [11–13]. An estimated 14–24% of GC/GEJC patients express PD-L1 in tumor cells, and an estimated 35% in the tumor microenvironment [14]. The KEYNOTE-061 trial reported combined positive scores (CPS) for PD-L1 expression, which includes expression on both tumor and immune cells. Among patients treated with PEM, 66.2% (*n* = 196) of patients expressed PD-L1 at a 1% CPS threshold [10].

The Cancer Genome Atlas Research Network (TCGA) evaluated 295 primary gastric adenocarcinomas for molecular characteristics and found that 22% had high microsatellite instability (MSI-H) [15]. However, there is variability based on other published estimates. A recent review estimates that 10–39% of gastroesophageal cancers are MSI-H [14], but the proportion seems to be considerably smaller in stage IV tumors [6, 10, 16, 17]. A recent prospective genomic profiling study of metastatic esophagogastric patients reported that only 3% of patients were MSI-H (*n* = 318) [16].

MSI-H GC patients show significantly higher PD-L1 expression, compared to MSS GC patients (25.9% versus 8.4%, *p*=0.003) [18, 19] and accumulating data suggest improved survival and durable response to immunotherapy among patients with MSI-H status. The KEYNOTE-059 and KEYNOTE-061 trials report overall response rates (ORR) of 57.1% and 46.7%, respectively, although the numbers of MSI-H patients were small (*n* = 7 and *n* = 15, respectively) [6, 10]. Despite the small sample sizes, these outcomes are consistent with a pooled ORR to PEM of 53.0% across different cancer sites for dMMR tumors (*n* = 86) [9].

Our analysis aimed to evaluate the impact of biomarker-based 2L GC treatment, compared to other commonly prescribed treatment regimens. In the biomarker-based strategies, patients with MSI-H/dMMR or PD-L1 positivity received PEM therapy, and the remaining patients received either PAC monotherapy or RAM/PAC combination therapy. We also evaluated best supportive care (BSC), PAC, PEM, and RAM/PAC for all GC patients on 2L treatment, regardless of biomarker status. The primary outcomes were QALYs as a measure of clinical effectiveness and incremental cost-effectiveness ratios (ICERs).

## 2. Methods

We utilized the CHEERS checklist for standardized methodology and reporting of cost-effectiveness analyses (Supplementary [Supplementary-material supplementary-material-1]).

### 2.1. Patient Characteristics

Our model simulated a hypothetical cohort of patients, which was constructed using pooled patient characteristics from the KEYNOTE-059, KEYNOTE-061, REGARD, and RAINBOW trials (Supplementary [Supplementary-material supplementary-material-1]) [6, 7, 10, 20]. The modeled cohort contained 60 year olds (70% male, 30% female) with unresectable recurrent/metastatic gastric adenocarcinomas undergoing second-line (2L) therapy. In accordance with the patient populations in the clinical trials, gastric adenocarcinomas include proximal, distal, and gastroesophageal junction (GEJ) cancers. The cohort entered the model following disease progression on a previous line of chemotherapy with a platinum and/or fluoropyrimidine, as well as trastuzumab in HER2 + patients. For the base case, we assumed that 40% of patients expressed PD-L1 at a CPS > 1% threshold and 10% of patients were MSI-H. These proportions were estimated based on proportions in the clinical trials and the literature [6, 10, 14–17, 21].

### 2.2. Treatment Model

**Figure 1:**
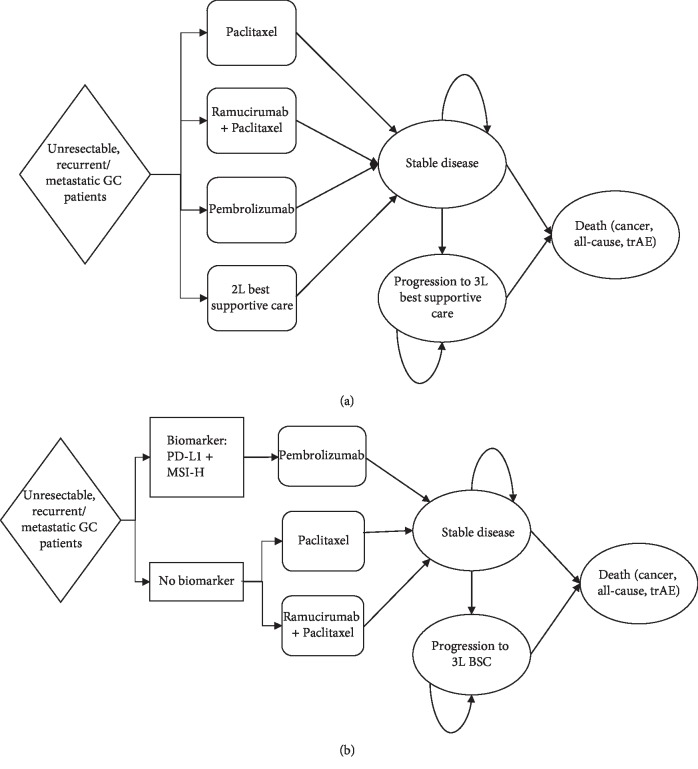
Model schematic. The top plot (a) illustrates treatment strategies with all patients receiving the same treatment, and the bottom plot (b) illustrates personalized treatment based on biomarker status. The biomarkers are programmed death ligand-1 (PD-L1) with a combined positive score (CPS) >1%, or high microsatellite instability (MSI-H).

A decision-analytic Markov model (Figure 1) was constructed in Python to analyze 2L treatments for GC patients. Patients could remain stable, experience disease progression, and die from cancer, severe adverse drug reactions, or age- and sex-specific all-cause mortality. Patients could present with treatment-related adverse events (trAEs) within the first 6 months of the model. After disease progression, patients were placed on best supportive care (BSC) as defined by the NCCN [5]. The model simulated a 60-month treatment and follow-up window, or 5-year time horizon, in monthly cycles. This time horizon was selected to capture the potential durable response to immunotherapy suggested in the literature [9, 16].

We compared eight unique treatment strategies that are endorsed by current NCCN guidelines and also recently studied within clinical trials [5]. In the first four strategies, all patients received the same treatment, regardless of biomarker status. These strategies were (1) BSC with no active treatment, (2) PEM monotherapy (200 mg flat dose or 2 mg/kg every three weeks), (3) PAC monotherapy (80 mg/m^2^ three times per month), and (4) combination therapy with PAC (80 mg/m^2^ three times per month) and RAM (8 mg/kg; every two weeks). In the remaining four strategies, treatment was based on biomarker status: MSI/dMMR or PD-L1 CPS of 1%. Patients with a positive biomarker status received PEM, and the rest received either PAC monotherapy or RAM/PAC combination therapy.

### 2.3. 2L Cancer Progression and Mortality Estimates

Rates of disease progression and cancer mortality in each strategy were derived from the Kaplan–Meier (KM) estimates of overall survival (OS) and progression-free survival (PFS) of the respective clinical trials using piecewise linear fits to the curves (Table 1). Supplementary [Supplementary-material supplementary-material-1] compares model outputs to clinical trial data. The PEM arms (including whole-population, MSI-H, and PD-L1 + subsets) were informed by data from the phase 3 open-label KEYNOTE-061 trial on pretreated gastric and GEJ adenocarcinomas [10]. PFS for the MSI-H subset was modeled using pooled data for all noncolorectal cancer sites [9]. Second-line treatment with PAC monotherapy and RAM/PAC was informed by data from the double-blind randomized phase 3 RAINBOW trial comparing the two treatments [7]. The BSC arm was informed by data from the placebo plus BSC group of the REGARD trial [20]. The same data were used to estimate the probability of cancer-specific mortality after disease progression and discontinuation of treatment in all strategies.

Age- and sex-specific all-cause mortality were estimated using 2014 US data from the Centers for Disease Control [40]. To be consistent with the patient population in the clinical trials, the probability of all-cause mortality was a weighted sum of male and female mortality data in a 70 : 30 ratio. When needed, the OS and PFS data were extrapolated beyond the time horizon of the clinical trials (∼24 months) by extending the KM curves and all-cause mortality rates.

### 2.4. Treatment-Related Adverse Events

The monthly probabilities of treatment-related adverse events (trAE) and related death or treatment discontinuation associated with each strategy were estimated as weighted averages from the safety data of the respective trials, namely, the KEYNOTE-012, KEYNOTE-059, KEYNOTE-061, and KEYNOTE-006 (melanoma) trials on PEM and the RAINBOW trial on RAM/PAC (Table 1) [6, 7, 10, 20, 30, 31]. These probabilities were multiplied by the proportion of patients receiving treatment in each cycle (i.e., patients with stable disease). Associated costs and disutilities were then applied based on the resulting percentages. We assumed that patients who discontinued treatment entered the progressive disease state. In addition, we assumed that trAEs occurred within the first six months of treatment based on estimates of the timing of trAEs in the literature [31, 41]. Late-onset trAEs were neglected due to insufficient data. We also assumed that the toxicity profile for PEM remained consistent across biomarker subgroups. Finally, we accounted for differences in hospitalization rates across treatment arms by modifying the percentage of the population experiencing a grade 3 or 4 trAE, and assuming that only this portion of the population would be eligible for inpatient admission.

### 2.5. Estimates of Costs and Quality of Life

Cost and quality of life model inputs are summarized in Table 1. We estimated costs of biomarker testing, drug acquisition and administration, and follow-up visits to general practitioners, oncologists, and radiologists from a US payer perspective. The costs of biomarker testing were estimated using the 2019 CMS Physician Fee Schedule and Clinical Diagnostic Laboratory Fee Schedule [23, 24]. Drug costs were estimated using the 2019 Medicare Average Sales Price Drug Pricing File, assuming an average patient weight of 65 kg and average body surface area of 1.85 m^2^ (weighted average for men and women in a 70 : 30 ratio to reflect clinical trial populations) [32, 42, 43]. Drug administration costs were derived from the literature and adjusted to reflect the number of infusions administered per month, multiple therapies, and associated costs of premedication [33].

The monetary costs associated with trAEs were assumed to be primarily those resulting from hospitalization costs. These were estimated as weighted averages of the costs associated with specific grades 3-4 trAEs and the frequency of occurrence of those events in the respective trials [22, 33, 34, 44, 45]. Hospitalization due to trAEs was assumed to occur for grades 3-4 only, at a rate that was estimated using insurance claims data [4]. Costs associated with trAEs managed in outpatient settings were assumed to be negligible. All costs were in US dollars (USD).

Quality-adjusted life years (QALYs) were derived by adjusting survival times by health state utility scores (0 = death, 1 = perfect health) as measures of quality of life (QoL). Utility scores associated with PFS and 3L BSC following progression were estimated from the literature and adjusted by the distributions of ECOG scores in clinical trials [25, 26]. Published estimates report utility score for a patient with an ECOG score of 1 is 0.1 less than the utility score of a patient with an ECOG score of 0. Reductions in QoL due to grades 1-2 and 3-4 trAEs were accounted for using disutility scores from a previous study on nivolumab for colorectal cancer [36]. As with costs, disutilities for trAEs were estimated as weighted averages for each treatment arm. All costs and utility scores were discounted by 3% annually [46].

### 2.6. Model Outcomes

The primary outcomes of the analysis were QALYs gained and ICERs, analyzed in the context of an efficiency frontier. ICERs were calculated as the ratio of differences in costs and QALYs between a strategy and the next best alternative. A willingness-to-pay (WTP) threshold of $100,000 per QALY gained was used in the base case as the cutoff for cost effectiveness [47]. Secondary outcomes were survival (unadjusted life expectancy) and total costs.

### 2.7. Sensitivity Analysis

We quantified uncertainty by measuring model output variation in response to changing input parameters via deterministic (one-way) and probabilistic sensitivity analyses (PSA). We focused on the ICER endpoint in these analyses in order to determine if cost-effectiveness results would change when changing input parameters. One-way sensitivity analyses were performed by varying one parameter at a time within prescribed bounds and recording the change in ICERs in a tornado diagram. In probabilistic sensitivity analyses, all parameters were sampled simultaneously from probability distributions in 10000 Monte Carlo samples per strategy, and the outcomes were recorded into cost-effectiveness planes. The ranges for the one-way sensitivity analyses and the distributions for the probabilistic sensitivity analyses are presented in Table 2, and detailed methods are given in the Supplementary Materials. Ranges for cancer mortality and cancer progression rates for MSI-H patients on PEM are depicted as ranges in KM curves in Supplementary [Supplementary-material supplementary-material-1].

## 3. Results

### 3.1. Base Case

**Figure 2:**
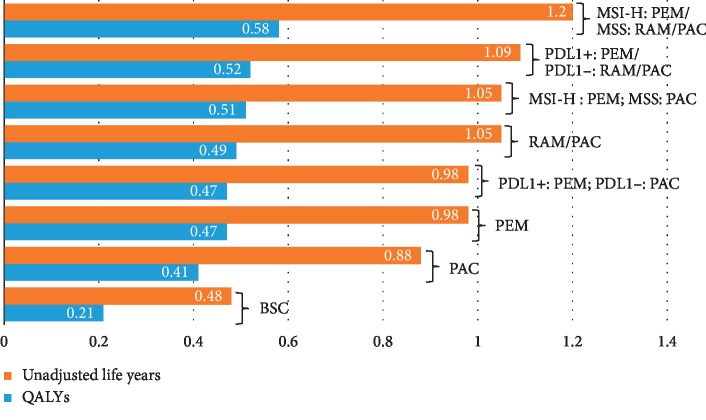
Unadjusted life years and quality-adjusted life years (QALYs). This graph illustrates the base case results in terms of total unadjusted life years and QALYs.

**Figure 3:**
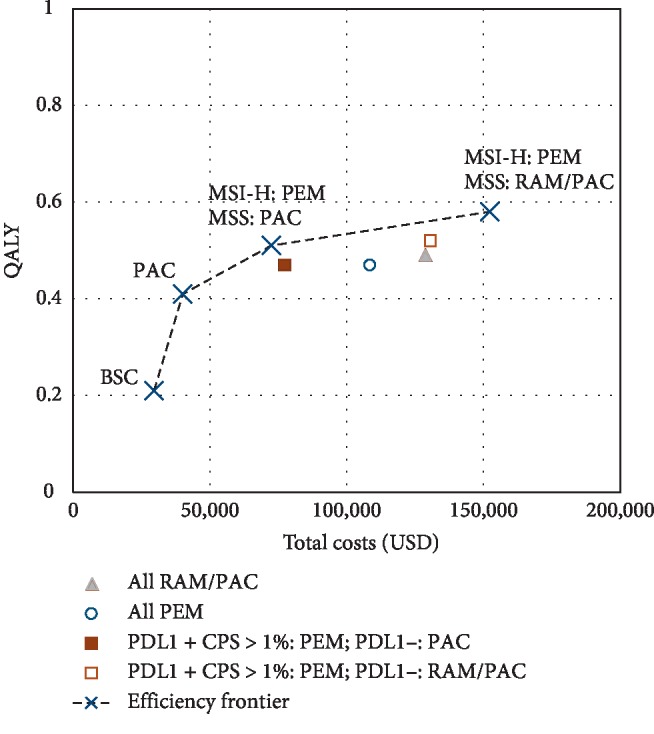
Efficiency frontier. This plot represents the base case results in terms of quality-adjusted life years (QALYs) and costs.

The base case results for all strategies are presented in Table 3, Figures 2 and 3 (efficiency frontier). Overall survival and progression-free survival for strategies on the efficiency frontier are shown in Supplementary [Supplementary-material supplementary-material-1]. Biomarker-based strategies substantially increased quality-adjusted survival and unadjusted survival compared to BSC and PAC (Figure 2). All biomarker-based strategies produced similar QALY outputs, between 0.47 and 0.58 QALYs or a variation of only 1.32 months. The most effective strategy was PEM for MSI-H and RAM/PAC for MSS patients, with a total of 1.2 life years and 0.58 QALYs. This strategy increased unadjusted survival by 8.6 months compared to BSC and 3.8 months compared to PAC. In addition, QALYs increased by 4.4 months compared to BSC and 2.0 months compared to PAC.

The effectiveness of biomarker-based strategies improved cost effectiveness, but the ICERs still surpassed the WTP threshold. PAC for all patients was the only cost-effective strategy, with an ICER of $53705/QALY. PEM for MSI-H patients and PAC for MSS patients resulted in an ICER of $325611/QALY, and PEM for MSI-H patients and RAM/PAC for MSS patients resulted in an ICER of $1074620/QALY.

### 3.2. Sensitivity Analyses

**Figure 4:**
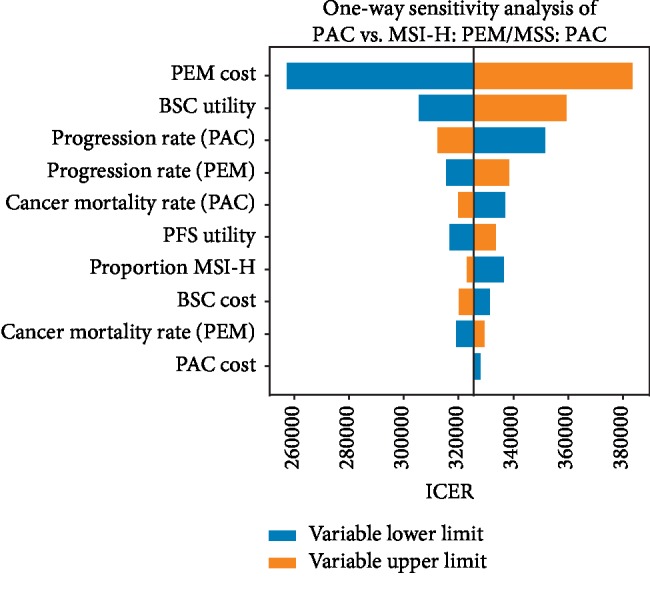
One-way sensitivity analysis. This tornado diagram illustrates the results of a one-way sensitivity analysis of key input parameters. The ranges of incremental cost-effectiveness ratios (ICERs) for the MSI-H: PEM/MSS: PAC strategy compared to the PAC for all patients' strategy are plotted.

Deterministic and probabilistic sensitivity analyses indicated that our base case results were robust to uncertainty in model parameters. The tornado diagram in Figure 4 illustrates the results of a one-way sensitivity analysis for the biomarker-based strategy with the lowest ICER: PEM for MSI-H patients and PAC for MSS patients. The cost of PEM had the greatest effect on model outcomes. However, MSI-H: PEM/MSS: PAC was not cost-effective for any costs tested in the sensitivity analyses. Supplementary [Supplementary-material supplementary-material-1] illustrates the results of the probabilistic sensitivity analysis for two main strategies on the efficiency frontier: (1) PAC versus BSC and (2) MSI-H: PEM/MSS: PAC versus PAC for all patients. In 96% of iterations, PAC remained a cost-effective strategy. In 87% of iterations, MSI-H: PEM/MSS: PAC was not cost-effective.

Total costs and total QALYs increased with MSI-H prevalence, resulting in total costs between $47000 and $111000, and total QALYs between 0.43 and 0.63 (Supplementary [Supplementary-material supplementary-material-1]). However, the ICERs remained quite stable since biomarker prevalence drove both costs and QALYs.

## 4. Discussion

Our analysis used a decision-analytic framework to analyze the impact of personalized, biomarker-based regimens for second-line treatment of metastatic gastric cancer. The most effective treatment regimens were biomarker-based, with PEM for MSI-H patients and RAM/PAC for MSS patients resulting in the most QALYs. Our study highlights the benefit of the broader ongoing shift toward personalized, biomarker-based cancer treatment in patients who would have been offered supportive/palliative care in the past. Historically, only about 20% of GC patients went on to 2L therapy, but, with increasing efficacy in the 2L setting, that number has increased to over 50% [4, 48].

Perhaps reflecting the diversity of current treatment, the NCCN recommendations for this patient population encompass a broad range of approaches, including basic supportive care. Although our modeling analysis finds substantial benefit for targeted treatment compared to supportive care or conventional chemotherapy (paclitaxel), these newer strategies are expensive and not cost-effective. Improving the precision of predictive biomarkers of response and reducing the cost of immunotherapeutic and biologic agents could continue to improve the cost effectiveness of targeted therapies.

Our analysis provides important data for decision-making, as clinical trials directly comparing such a large number of potential treatment strategies are not feasible. As a model-based analysis, we were able to incorporate quality of life and cost considerations to estimate outcomes that are of interest to various stakeholders. We also developed our model utilizing the most current data available and provided extensive details regarding our approach and assumptions to provide transparency and diminish concerns about unknown biases in the model (see additional details in Supplementary Materials). Finally, we performed extensive sensitivity analyses to explore the impact of uncertainty in model parameters on analysis results.

We identified several limitations in the current study. In the absence of long-term follow-up data, we extended the Kaplan–Meier estimators of overall and progression-free survival beyond the endpoints of the associated clinical trials. In addition, we used data for all patients to inform the survival outcomes for MSS/PD-L1 patients receiving PAC or RAM/PAC. Because our model inputs were derived from clinical trial data collected from patients with high-performance status, the generalizability of results to those with limited performance may be limited. Nonetheless, patient characteristics across clinical trials were comparable (see Supplementary Materials). Finally, late-onset severe adverse events associated with targeted therapies and immunotherapies were not included in the model due to insufficient data.

Our analysis was based on currently available biomarkers and treatments. Biomarker discovery is an active area of investigation and future biomarkers that improve the precision of treatment selection could improve both the effectiveness and cost-effectiveness of novel strategies. Although the biomarker-based approach we assessed in our current analysis did not meet the willingness-to-pay threshold for cost effectiveness, the ability to further define the optimal treatment group might continue to reduce the ICER to a point where these therapies achieve cost effectiveness.

Primary drivers of the cost-effectiveness results were the current costs of newer therapeutic agents. The most effective strategies were expensive, rendering those strategies cost-ineffective. A threshold analysis indicated that the cost of PEM would have to be approximately $3200 per month in order for the MSI-H: PEM/MSS: PAC strategy to be cost-effective (Supplementary [Supplementary-material supplementary-material-1]). The cost of cancer is a serious matter, especially as more effective and more costly treatments enter the market. Emerging research has found high (28%–48%) rates of treatment-related financial toxicity in cancer patients, with negative consequences for patient-reported outcomes and treatment adherence [49]. A combination of improving precision medicine and lowering drug prices could achieve cost effectiveness and improve patient outcomes. Future clinical data will allow for improved model inputs and fewer assumptions than those made for the current analysis.

In conclusion, our modeling analysis finds that personalized, biomarker-based treatments improve both quality-adjusted and quality-unadjusted survival but are not cost-effective, largely because of the prices of therapeutic agents.

## Figures and Tables

**Table 1:** Summary of model inputs. Costs, probabilities, and utilities are per month unless otherwise specified.

Parameter type	Parameter	Base case estimate
*All treatment arms*		
Probabilities	Cancer-specific mortality on 3L best supportive care	0.1476 [20]
	trAE-related hospitalization	0.0440 [4]
Costs	Cost of GP follow-up	$179.39 [22]
	Cost of oncology follow-up	$355.35 [22]
	Cost of radiology follow-up	$284.00 [22]
	One-time cost: PD-L1 testing (CPT 88342)	$118.51 [23]
	One-time cost: MSI-H testing (CPT 81301)	$348.56 [24]
Utilities	2L progression-free survival	0.622 [25, 26]
	3L best supportive care	0.362 [25, 26]

*Best supportive care*		
Probabilities	Disease progression
	Months 0–3	0.414 [20]
	Months 4–10	0.347 [20]
	Months 11+	0.251 [20]
	Cancer-specific mortality	0.129 [20]
Costs	Average cost of routine home care Medicare rate (2018)	$5169.60 [27, 28]

*Pembrolizumab*		
Probabilities	Disease progression: biomarker agnostic
	Months 0–1	0.31 [10]
	Months 2–9	0.122 [10]
	Months 10+	0.014 [10]
	Cancer-specific mortality	0.01857 [10]
	Disease progression: PDL1 + CPS > 1%
	Months 0–3	0.2940 [10]
	Months 4–10	0.0998 [10]
	Months 11+	0.0127 [10]
	Cancer-specific mortality	0.01179 [10]
	Disease progression: MSI-H
	Months 0–3	0.0835 [10, 29]
	Months 4–10	0.0249 [10, 29]
	Months 11+	0.0134 [10, 29]
	Cancer-specific mortality	0.0057 [10]
	Adverse events
	Grade 1/2	0.1035 [6, 7, 10, 30, 31]
	Grade 3/4	0.0237 [6, 7, 10, 30, 31]
	Discontinuation	0.0028 [6, 7, 10, 30, 31]
	Death	0.00098 [6, 7, 10, 30, 31]
Costs	Pembrolizumab	$12246.75 [32]
	Drug administration	$470 [33]
	trAEs (grade 3/4)	$19487 [34, 35]
Disutilities	trAEs (grade 1/2)	−0.086 [36]
	trAEs (grade 3/4)	−0.165 [36]

*Paclitaxel*		
Probabilities	Disease progression
	Months 0–3	0.1870 [7]
	Months 4–10	0.1730 [7]
	Months 11+	0.1430 [7]
	Cancer-specific mortality	0.028 [7]
	Adverse events
	Grade 1/2	0.0864 [7, 10]
	Grade 3/4	0.0644 [7, 10]
	Discontinuation	0.0085 [7, 10]
	Death	0.0021 [7, 10]
Costs	Paclitaxel	$66.53 [32]
	Drug administration	$1412 [33]
	trAEs (grade 3/4)	$13198 [34, 35]
Disutilities	trAEs (grade 1/2)	−0.071 [36–39]
	trAEs (grade 3/4)	−0.180 [36–39]
*Ramucirumab* + *paclitaxel*		
Probabilities	Disease progression
	Months 0–1	0.1176 [7]
	Months 2–11	0.1843 [7]
	Months 12+	0.03297 [7]
	Cancer-specific mortality	0.01235 [7]
	Adverse events
	Grade 1/2	0.0306 [7]
	Grade 3/4	0.1773 [7]
	Discontinuation	0.0210 [7]
	Death	0.0034 [7]
Costs	Ramucirumab + paclitaxel	$12974 [32]
	Drug administration	$2118 [33]
	trAEs (grade 3/4)	$20658 [34, 35]
Disutilities	trAEs (grade 1/2)	−0.110 [36]
	trAEs (grade 3/4)	−0.250 [36]

**Table 2:** Summary of parameters for sensitivity analyses.

Parameter	Range (SD)	Distribution
*Proportion MSI-H*	0.02–0.22 (0.05)	Beta
Probabilities		
Probability of disease progression (MSI-H: PEM)	±30%	Uniform
Probability of disease progression (all other strategies)	±30%	Normal
Probability of cancer mortality on 3L best supportive care	±30%	Normal
*Costs (total)*		
Pembrolizumab	$10831–$15331 ($1354)	Gamma
Pembrolizumab trAEs (grade 3/4)	$14615–$24358 ($2436)	Gamma
Paclitaxel	$1843–$2343 ($230)	Gamma
Paclitaxel trAEs (grade 3/4)	$9898–$16497 ($1649)	Gamma
Ramucirumab + paclitaxel	$11996–$16996 ($1500)	Gamma
Ramucirumab + paclitaxel trAEs (grade 3/4)	$15493–$25822 ($2582)	Gamma
Best supportive care	$4136–$6136 ($311)	Gamma
*Utilities/Disutilities*		
2L progression-free survival	0.55–0.69	Uniform
3L best supportive care following progression	0.22–0.57	Uniform
Pembrolizumab trAEs (grade 1/2)	0.02–0.14	Uniform
Pembrolizumab trAEs (grade 3/4)	0.08–0.22	Uniform
Paclitaxel trAEs (grade 1/2)	0.05–0.13	Uniform
Paclitaxel trAEs (grade 3/4)	0.06–0.37	Uniform
Ramucirumab + paclitaxel trAEs (grade 1/2)	0.00–0.24	Uniform
Ramucirumab + paclitaxel trAEs (grade 3/4)	0.16–0.35	Uniform

**Table 3:** Summary of base case results.

Treatment scenario		Cost (USD)	Unadjusted life years	QALY	ICER
1	BSC, all	29614	0.48	0.21	––
2	PAC, all	40108	0.88	0.41	53705
4	PEM all	108408	0.98	0.47	Dominated
5	PDL1 + (CPS > 1%): PEM PDL1-: PAC	77359	0.98	0.47	Dominated
3	RAM/PAC all	128775	1.05	0.49	Dominated
6	MSI-H: PEM MSS: PAC	72442	1.05	0.51	325611
7	PDL1 + (CPS > 1%): PEM PDL1-: RAM/PAC	130560	1.09	0.52	Weakly dominated
8	MSI-H: PEM MSS: RAM/PAC	152242	1.2	0.58	1074620

## Data Availability

The cost and effectiveness data used to support the findings of this study are included within the article with citations to prior studies (Table 3). The source code for the model is available from the corresponding author upon request.

## Supplementary Materials

Supplementary Materials The supplementary materials provide details on additional methods: sensitivity analyses. Supplementary Table 1: summary of clinical trials used to inform rates of disease progression and cancer mortality. GEJ: gastroesophageal junction; ECOG: Eastern Cooperative Oncology Group. Supplementary Figure 1: comparison of model outputs (lines) and clinical trial data (points) for each treatment and selection of biomarker populations. Supplementary Figure 2: comparison of model outputs for overall survival (a) and progression-free survival (b) among select strategies. Supplementary Figure 3: Kaplan–Meier (KM) curves illustrating sensitivity analysis bounds on (a) cancer mortality rates (OS curve) and (b) cancer progression rates (PFS curve). Both curves represent MSI-H PEM data. Supplementary Figure 4: results of MSI-H prevalence one-way sensitivity analysis: changes in (a) total costs and (b) total QALYs for the MSI-H: PEM/MSS: PAC treatment strategy. Supplementary Figure 5: threshold analysis to determine the cost of PEM needed to achieve cost effectiveness of the MSI-H: PEM/MSS: PAC strategy. Supplementary Figure 6: results of the probabilistic sensitivity analyses for the two main strategies on the efficiency frontier. Supplementary Table 2: CHEERS checklist [11].
