# Supplementary material for: Cost-Effectiveness Analysis of Biomarker-Guided Treatment for Metastatic Gastric Cancer in the Second-Line Setting
Source: J Oncol. 2020 Feb 17;2020:2198960. doi: 10.1155/2020/2198960 (PMC7048937; doi:10.1155/2020/2198960)
Supplement: Supplementary Materials — The supplementary materials provide details on additional methods: sensitivity analyses. Supplementary Table 1: summary of clinical trials used to inform rates of disease progression and cancer mortality. GEJ: gastroesophageal junction; ECOG: Eastern Cooperative Oncology Group. Supplementary Figure 1: comparison of model outputs (lines) and clinical trial data (points) for each treatment and selection of biomarker populations. Supplementary Figure 2: comparison of model outputs for overall survival (a) and progression-free survival (b) among select strategies. Supplementary Figure 3: Kaplan–Meier (KM) curves illustrating sensitivity analysis bounds on (a) cancer mortality rates (OS curve) and (b) cancer progression rates (PFS curve). Both curves represent MSI-H PEM data. Supplementary Figure 4: results of MSI-H prevalence one-way sensitivity analysis: changes in (a) total costs and (b) total QALYs for the MSI-H: PEM/MSS: PAC treatment strategy. Supplementary Figure 5: threshold analysis to determine the cost of PEM needed to achieve cost effectiveness of the MSI-H: PEM/MSS: PAC strategy. Supplementary Figure 6: results of the probabilistic sensitivity analyses for the two main strategies on the efficiency frontier. Supplementary Table 2: CHEERS checklist [11]. [file 2198960.f1.docx]

**SUPPLEMENTARY MATERIALS**

Cost-effectiveness analysis of biomarker-guided treatment for metastatic gastric cancer in the second-line setting.

Brianna Lauren; Sassan Ostvar; Elisabeth Silver; Myles Ingram; Aaron Oh; Lindsay Kumble; Monika Laszkowska; Jacqueline N. Chu; Dawn L. Hershman; Gulam Manji; Alfred I. Neugut; Chin Hur.

Author Affiliations: Columbia University Medical Center, New York, NY (Ostvar, Lauren, Silver, Ingram, Oh, Kumble, Laszkowska, Hershman, Manji, Hur); Columbia University Irving Cancer Research Center (Hershman, Manji, Neugut, Hur); Massachusetts General Hospital, Boston, MA (Chu)

**Corresponding Author:**
Chin Hur, MD, MPH
Columbia University Medical Center
622 W 168^th^ Street
PH9-105
New York, NY 10032
212-305-2020
[ch447@cumc.columbia.edu](mailto:ch447@cumc.columbia.edu)

**Additional methods: Sensitivity analyses**

The ranges for the one-way sensitivity analyses and the distributions for the probabilistic sensitivity analyses are presented in Table 2 of the main text. Given the uncertainty in the prevalence of MSI-H in our patient population, we tested values between 2% and 22%. In the probabilistic sensitivity analysis (PSA), the proportion of MSI-H patients was sampled from a beta distribution, with a mean of 0.1 (base case) and a standard deviation of 0.05. This distribution is skewed right in accordance with literature suggesting a lower proportion of MSI-H patients for this patient population [1-4].

Probabilities associated with disease progression and cancer mortality were sampled within ±30% of the base case KM estimate (means). We used normal distributions for most strategies. However, we anticipated the largest uncertainty to exist in the strategies using PEM for the MSI-H subgroup. Effectiveness data for this arm was available for a small group of patients (n=15) [2], and there is a great deal of heterogeneity in the estimates for the clinical presentation of microsatellite instability [3-8]. For this reason, we used a uniform distribution to determine percent change in these transition probabilities, as outlined in Table 2 of the main text. Supplementary Figure 2 illustrates the ranges of KM curves resulting from this sampling.

The range of costs represented ±20% of the base case values. In the PSA, costs were sampled from gamma distributions, with standard deviations obtained from the literature or, if unavailable, equal to 10% of the base case values. Ranges for utility values were obtained from the literature and sampled from uniform distributions in the PSA. We used uniform distributions because of the high uncertainty for utility values.

**Supplementary Table 1:** Summary of clinical trials used to inform rates of disease progression and cancer mortality. GEJ: gastroesophageal junction; ECOG: Eastern Cooperative Oncology Group

|  | **REGARD** | **RAINBOW** | **KEYNOTE-061** |
| --- | --- | --- | --- |
| **Treatment** | Ramucirumab vs. best supportive care | Ramucirumab plus paclitaxel vs. placebo plus paclitaxel | Pembrolizumab vs. paclitaxel |
| **Number of participants** | 355 | 665 | 592 |
| **Gastric:GEJ cancer ratio** | 74:26 | 80:20 | 70:30 |
| **Line of therapy** | 2^nd^ | 2^nd^ | 2^nd^ |
| **Average age** | 60 | 61 | 61 |
| **Male:Female ratio** | ~70:30 | ~70:30 | ~70:30 |
| **Median overall survival** | 3.8 months (BSC) | 9.6 months (RAM/PAC);  7.4 months (PAC) | 9.1 months (PEM) |
| **0:1 ECOG score ratio** | 26:73 (BSC) | 35:65 (RAM/PAC)  43:57 (PAC) | 43:57 (PEM) |
| **Citation** | [9] | [10] | [2] |

**Supplementary Figure 1:** Comparison of model outputs (lines) and clinical trial data (points) for each treatment and select biomarker populations.

**
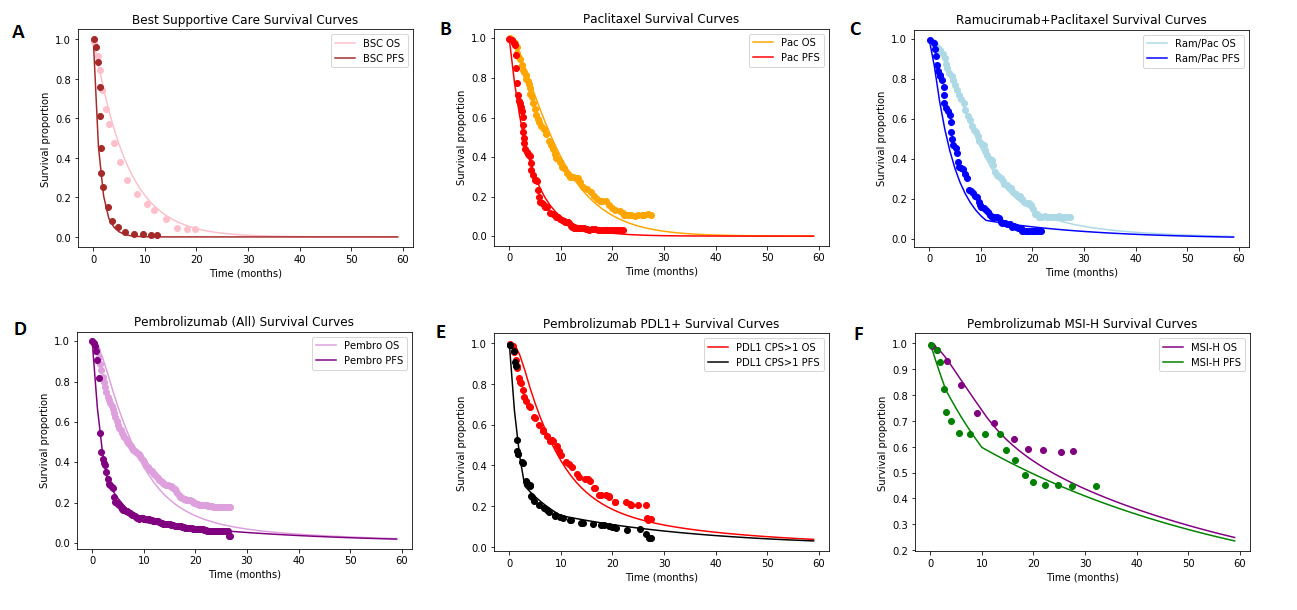
**

**Supplementary Figure 2:** Comparison of model outputs for overall survival (A) and progression-free survival (B) among select strategies.

***
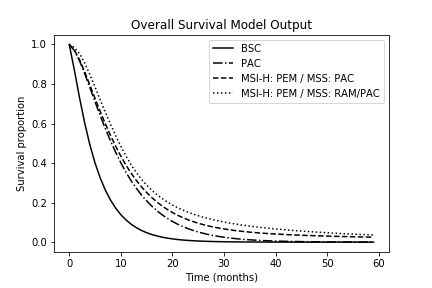

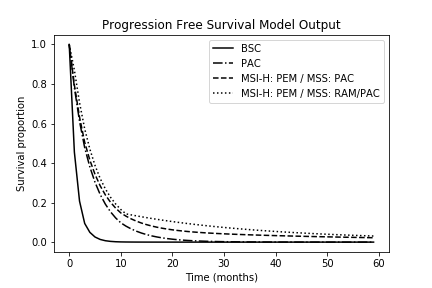
***

**B**

**A**

**Supplementary Figure 3:** Kaplan Meier (KM) curves illustrating sensitivity analysis bounds on (A) cancer mortality rates (OS curve), and (B) cancer progression rates (PFS curve). Both curves represent MSI-H PEM data.

**B**

**A**


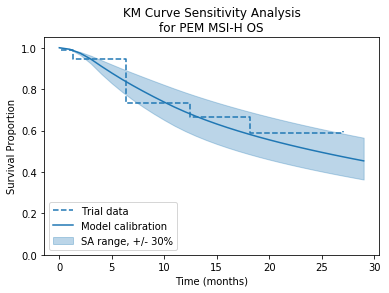

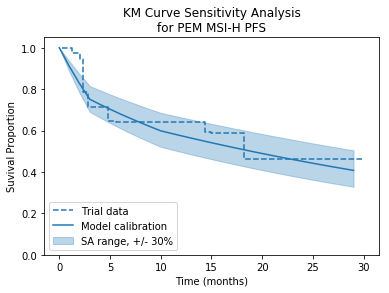


**Supplementary Figure 4:** Results of MSI-H prevalence one-way sensitivity analysis: changes in (A) total costs and (B) total QALYs for the MSI-H: PEM / MSS: PAC treatment strategy.


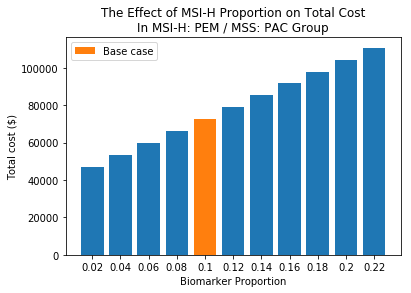
 
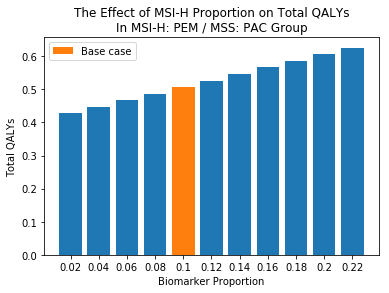


**A**

**B**

**Supplementary Figure 5:** Threshold analysis to determine the cost of PEM needed to achieve cost-effectiveness of the MSI-H: PEM / MSS: PAC strategy.


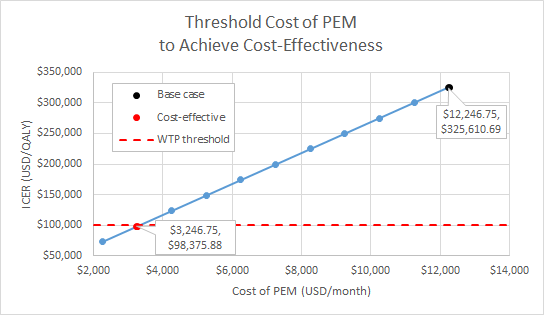


**Supplementary Figure 6:** Results of the probabilistic sensitivity analyses for the two main strategies on the efficiency frontier.


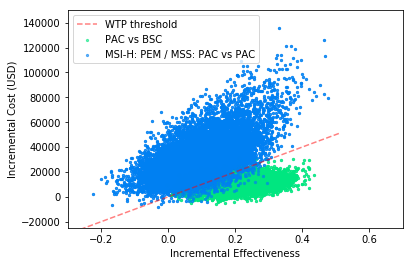


**Supplementary Table 2 - CHEERS checklist [11]**

| Section/Item | Item No. | Recommendation | Reported? (Page) |
| --- | --- | --- | --- |
| **Title and abstract** | | | |
| Title | 1 | Identify the study as an economic evaluation or use more specific terms such as “cost-effectiveness analysis”, and describe the interventions compared. | ✓ (1) |
| Abstract | 2 | Provide a structured summary of objectives, perspective, setting, methods (including study design and inputs), results (including base case and uncertainty analyses), and conclusions. | ✓ (2-3) |
| **Introduction** | | | |
| Background and objectives | 3 | Provide an explicit statement of the broader context for the study. | ✓ (4) |
|  |  | Present the study question and its relevance for health policy or practice decisions. | ✓ (4-6) |
| **Methods** | | | |
| Target population and subgroups | 4 | Describe characteristics of the base case population and subgroups analyzed, including why they were chosen. | ✓ (6-7) |
| Setting and location | 5 | State relevant aspects of the system(s) in which the decision(s) need(s) to be made. | ✓ (6-7) |
| Study perspective | 6 | Describe the perspective of the study and relate this to the costs being evaluated. | ✓ (9) |
| Comparators | 7 | Describe the interventions or strategies being compared and state why they were chosen. | ✓ (7-8) |
| Time horizon | 8 | State the time horizon(s) over which costs and consequences are being evaluated and say why appropriate. | ✓ (7) |
| Discount rate | 9 | Report the choice of discount rate(s) used for costs and outcomes and say why appropriate. | ✓ (10) |
| Choice of health outcomes | 10 | Describe what outcomes were used as the measure(s) of benefit in the evaluation and their relevance for the type of analysis performed. | ✓ (10) |
| Measurement of effectiveness | 11a | Single study-based estimates: Describe fully the design features of the single effectiveness study and why the single study was a sufficient source of clinical effectiveness data. | N/A |
|  | 11b | Synthesis-based estimates: Describe fully the methods used for identification of included studies and synthesis of clinical effectiveness data. | ✓ (7-9) |
| Measurement and valuation of preference-based outcomes | 12 | If applicable, describe the population and methods used to elicit preferences for outcomes. | N/A |
| Estimating resources and costs | 13a | Single study-based economic evaluation: Describe approaches used to estimate resource use associated with the alternative interventions. Describe primary or secondary research methods for valuing each resource item in terms of its unit cost. Describe any adjustments made to approximate to opportunity costs. | N/A |
|  | 13b | Model-based economic evaluation: Describe approaches and data sources used to estimate resource use associated with model health states. Describe primary or secondary research methods for valuing each resource item in terms of its unit cost. Describe any adjustments made to approximate to opportunity costs. | ✓ (9-10) |
| Currency, price data, and conversion | 14 | Report the dates of the estimated resource quantities and unit costs. Describe methods for adjusting estimated unit costs to the year of reported costs if necessary. Describe methods for converting costs into a common currency base and the exchange rate. | ✓ (9-10) |
| Choice of model | 15 | Describe and give reasons for the specific type of decision-analytical model used. Providing a figure to show model structure is strongly recommended. | ✓ (7; Figure 1) |
| Assumptions | 16 | Describe all structural or other assumptions underpinning the decision-analytical model. | ✓ (7-10) |
| Analytical methods | 17 | Describe all analytical methods supporting the evaluation. This could include methods for dealing with skewed, missing, or censored data; extrapolation methods; methods for pooling data; approaches to validate or make adjustments (such as half cycle corrections) to a model; and methods for handling population heterogeneity and uncertainty. | ✓ (7-10) |
| **Results** | | | |
| Study parameters | 18 | Report the values, ranges, references, and, if used, probability distributions for all parameters. Report reasons or sources for distributions used to represent uncertainty where appropriate.  Providing a table to show the input values is strongly recommended. | ✓ (Tables 1-2; Supplementary Table 1) |
| Incremental costs and outcomes | 19 | For each intervention, report mean values for the main categories of estimated costs and outcomes of interest, as well as mean differences between the comparator groups. If applicable, report incremental cost-effectiveness ratios. | ✓ (Table 3) |
| Characterizing uncertainty | 20a | Single study-based economic evaluation: Describe the effects of sampling uncertainty for the estimated incremental cost and incremental effectiveness parameters, together with the impact  of methodological assumptions (such as discount rate, study perspective). | N/A |
|  | 20b | Model-based economic evaluation: Describe the effects on the results of uncertainty for all input parameters, and uncertainty related to the structure of the model and assumptions. | ✓ (12-13) |
| Characterizing heterogeneity | 21 | If applicable, report differences in costs, outcomes, or cost-effectiveness that can be explained by variations between subgroups of patients with different baseline characteristics or other observed  variability in effects that are not reducible by more information. | ✓ (11-13) |
| **Discussion** | | | |
| Study findings, limitations, generalizability, and current knowledge | 22 | Summarize key study findings and describe how they support the conclusions reached. Discuss limitations and the generalizability of the findings and how the findings fit with current knowledge. | ✓ (13-15) |
| **Other** | | | |
| Source of funding | 23 | Describe how the study was funded and the role of the funder in the identification, design, conduct, and reporting of the analysis. Describe other non-monetary sources of support. | ✓ (16) |
| Conflicts of interest | 24 | Describe any potential for conflict of interest of study contributors in accordance with journal policy. In the absence of a journal policy, we recommend authors comply with International Committee  of Medical Journal Editors recommendations. | ✓ (16) |

**Supplementary References**

1. Fuchs CS, Doi T, Jang RW-J*, et al.* KEYNOTE-059 cohort 1: Efficacy and safety of pembrolizumab (pembro) monotherapy in patients with previously treated advanced gastric cancer. Journal of Clinical Oncology 2017;35(15_suppl):4003-4003.

2. Shitara K, Özgüroğlu M, Bang Y-J*, et al.* Pembrolizumab versus paclitaxel for previously treated, advanced gastric or gastro-oesophageal junction cancer (KEYNOTE-061): a randomised, open-label, controlled, phase 3 trial. The Lancet 2018;392(10142):123-133.

3. Janjigian YY, Sanchez-Vega F, Jonsson P*, et al.* Genetic Predictors of Response to Systemic Therapy in Esophagogastric Cancer. Cancer discovery 2018;8(1):49-58.

4. Kim H, An JY, Noh SH*, et al.* High microsatellite instability predicts good prognosis in intestinal-type gastric cancers. Journal of Gastroenterology and Hepatology 2011;26(3):585-592.

5. Le DT, Uram JN, Wang H*, et al.* PD-1 Blockade in Tumors with Mismatch-Repair Deficiency. New England Journal of Medicine 2015;372(26):2509-2520.

6. Le DT, Durham JN, Smith KN*, et al.* Mismatch repair deficiency predicts response of solid tumors to PD-1 blockade. Science (New York, N.Y.) 2017;357(6349):409-413.

7. Jin Z, Yoon HH. The promise of PD-1 inhibitors in gastro-esophageal cancers: microsatellite instability vs. PD-L1. Journal of gastrointestinal oncology 2016;7(5):771-788.

8. Ratti M, Lampis A, Hahne JC*, et al.* Microsatellite instability in gastric cancer: molecular bases, clinical perspectives, and new treatment approaches. Cellular and Molecular Life Sciences 2018;75(22):4151-4162.

9. Fuchs CS, Tomasek J, Yong CJ*, et al.* Ramucirumab monotherapy for previously treated advanced gastric or gastro-oesophageal junction adenocarcinoma (REGARD): an international, randomised, multicentre, placebo-controlled, phase 3 trial. The Lancet 2014;383(9911):31-39.

10. Wilke H, Muro K, Van Cutsem E*, et al.* Ramucirumab plus paclitaxel versus placebo plus paclitaxel in patients with previously treated advanced gastric or gastro-oesophageal junction adenocarcinoma (RAINBOW): a double-blind, randomised phase 3 trial. Lancet Oncol 2014;15(11):1224-35.

11. Husereau D, Drummond M, Petrou S*, et al.* Consolidated Health Economic Evaluation Reporting Standards (CHEERS)—Explanation and Elaboration: A Report of the ISPOR Health Economic Evaluation Publication Guidelines Good Reporting Practices Task Force. Value in Health 2013;16(2):231-250.
